# Edible Insects: A New Sustainable Nutritional Resource Worth Promoting

**DOI:** 10.3390/foods12224073

**Published:** 2023-11-09

**Authors:** Mengjiao Li, Chengjuan Mao, Xin Li, Lei Jiang, Wen Zhang, Mengying Li, Huixue Liu, Yaowei Fang, Shu Liu, Guang Yang, Xiaoyue Hou

**Affiliations:** 1 Jiangsu Key Laboratory of Marine Bioresources and Environment, Jiangsu Ocean University, Lianyungang 222005, China; xydxhl@163.com (M.L.); foroei@163.com (Y.F.); jdliushu@163.com (S.L.); 2020000050@jou.edu.cn (G.Y.); 2 Co-Innovation Center of Jiangsu Marine Bio-Industry Technology, Jiangsu Ocean University, Lianyungang 222005, China; 3 College of Marine Food and Bioengineering, Jiangsu Ocean University, Lianyungang 222005, China; mcj15055202109@163.com (C.M.); 2021122077@jou.edu.cn (X.L.); 17802521856@163.com (L.J.); zw19975066385@163.com (W.Z.); 15961304993@163.com (M.L.); 4 College of Plant Protection, Nanjing Agricultural University, Nanjing 210095, China; huixue_liu@163.com; 5 Jiangsu Marine Resources Development Research Institute, Jiangsu Ocean University, Lianyungang 222005, China

**Keywords:** edible insects, new nutritional sources, sustainability, farming and processing, insect food promotion

## Abstract

Edible insects are a highly nutritious source of protein and are enjoyed by people all over the world. Insects contain various other nutrients and beneficial compounds, such as lipids, vitamins and minerals, chitin, phenolic compounds, and antimicrobial peptides, which contribute to good health. The practice of insect farming is far more resource-efficient compared to traditional agriculture and animal husbandry, requiring less land, energy, and water, and resulting in a significantly lower carbon footprint. In fact, insects are 12 to 25 times more efficient than animals in converting low-protein feed into protein. When it comes to protein production per unit area, insect farming only requires about one-eighth of the land needed for beef production. Moreover, insect farming generates minimal waste, as insects can consume food and biomass that would otherwise go to waste, contributing to a circular economy that promotes resource recycling and reuse. Insects can be fed with agricultural waste, such as unused plant stems and food scraps. Additionally, the excrement produced by insects can be used as fertilizer for crops, completing the circular chain. Despite the undeniable sustainability and nutritional benefits of consuming insects, widespread acceptance of incorporating insects into our daily diets still has a long way to go. This paper provides a comprehensive overview of the nutritional value of edible insects, the development of farming and processing technologies, and the problems faced in the marketing of edible insect products and insect foods to improve the reference for how people choose edible insects.

## 1. Introduction

In the past, edible insects were consumed as food in some developing countries to meet their food needs. However, with the trend of shrinking populations in many parts of the world in recent years, especially in Europe and Asia [1]. Insufficient food is no longer the main problem affecting human survival, and more and more people are concerned about nutrition and health. In recent years, the global obesity rate has been rising, and obesity may lead to a series of diseases such as diabetes, cardiovascular disease, and cancer, so how to alleviate obesity is an urgent problem worldwide [2]. Edible insects have attracted much attention because of their rich nutritional and health components, and studies have proved that the bioactive compounds present in edible insects have the potential effect of weight control [3]. *Tenebrio molitor*, *Hermetia illucens* and *Musca domestica* have potential hypolipidemic and anti-obesity effects [2]. Chitosan isolated from *Acheta domesticus* can bind lipids and has been shown to control body weight in pigs to exert anti-obesity effects [4].Furthermore, the consumption of insects can enhance human health by replenishing micronutrients or increase intestinal probiotic content to promote human intestinal health [5]. In addition, edible insects offer positive environmental and economic benefits regarding high feed conversion rates, low negative environmental impacts, and low land use [6,7]. Although essential for feeding humans, traditional animal husbandry and agricultural cultivation methods significantly release greenhouse gases, including methane (CH_4_), carbon dioxide (CO_2_), and nitrogen oxide (N_2_O) [8,9]. The consumption of insects as a sustainable alternative source of nutrition helps to minimize negative impacts on the environment and to meet the growing health and nutritional demands for food. A 2013 paper published by the Food and Agriculture Organization of the United Nations entitled Edible Insects: Future Prospects for Food and Nutritional Security suggests that insects have the potential to become one of the staple foods of the future.

Eating insects has been practiced throughout human history. In ancient Greece, Aristotle even mentioned the enjoyment of eating cicadas in his book Animal History [10]. However, with the development of society and culture, the current traditional practice of consuming insects is declining in many regions [11]. Nevertheless, edible insects have recently gained renewed recognition for their high protein content, active substance, low environmental impact, and sustainability. Extensive research has shown that insects can be a valuable and innovative source of nutrition. Not only are they a wealthy source of protein and essential nutrients, but they also offer significant sustainability advantages over traditional agriculture and livestock farming practices. Compared to conventional livestock sources, obtaining nutrition from edible insects has several benefits. Insects have a small ecological footprint, as they require less land and water to thrive. Additionally, their growth cycles are rapid, and they don’t contribute to the depletion of arable land that is often necessary for animal farming. Therefore, embracing edible insects as a food source can help alleviate environmental pressures while ensuring a sustainable and nutritious future. Insects convert low-protein feed into edible protein very efficiently, and their small size results in low emissions of environmentally hazardous greenhouse gases and ammonia [12,13]. Comparatively, it takes eight times more land to obtain one gram of edible protein from beef than from mealworms. Mealworms (*Tenebrio molitor*) also have a smaller environmental impact in terms of greenhouse gas emissions compared to conventional animal agriculture. When considering edible protein, broiler chickens emit 32–167% more CO_2_ than mealworms, and beef cattle emit 6–13 times more CO_2_ than mealworms [14].

However, using insects as a sustainable food source requires advanced farming techniques to ensure consistent production, as well as sophisticated processing methods to ensure food quality and safety. In addition, market acceptance and the establishment of relevant laws and regulations are also challenges that need to be addressed for the widespread adoption of insect food. Cultural differences in attitudes towards edible insects further complicate the promotion of insect food. Ensuring insect food quality and safety requires a comprehensive regulatory system.

This review aims to explore in depth the multifaceted potential and advantages of edible insects. We will give a detailed analysis of the nutritional value of insects, the process of their utilization, and the application of insects in food, the sustainability of insects and their market acceptance and challenges in different cultures, and the regulatory issues facing insect utilization. Through these comprehensive analyses, we hope to provide fresh insights into the significant role that insects can play in the future of food. They have the potential to meet global nutritional needs and contribute to sustainable food production.

## 2. Nutritional Value and Functional Composition of Edible Insects

Edible insects contain high-quality protein, fat, vitamins, minerals, fiber, and some trace elements, and their nutritional benefits have been increasingly acknowledged [15]. In fact, they are comparable to, if not better than, conventional foods in terms of certain nutrients [16,17]. For example, the protein content of fresh edible caterpillars and chicken is 28 g/100 g fresh weight and 21 g/100 g fresh weight, respectively [18,19]. Furthermore, regarding digestibility, insect protein is comparable to milk, soya, and casein [20]. Commonly consumed insects can provide no less nutrition than conventional poultry (Table 1). A study conducted by Halloran et al. found that edible insects not only fulfill daily energy and nutrient requirements, but also contain essential amino acids, polyunsaturated and monounsaturated fatty acids, zinc, iron, and fiber [21]. Therefore, this makes edible insect proteins, fats, and other nutrients a viable alternative to traditional sources [22]. In addition to these traditional nutrients, insects also contain functional components like chitin, phenols, antioxidants, and antimicrobial peptides, which have potential benefits for immune support and disease prevention in humans.

### 2.1. Insect Proteins

Insects are particularly rich in high-quality proteins, surpassing those found in traditional livestock animals. Insect protein’s amino acid profile covers a wide range of essential amino acids required by humans, making it highly bioavailable and beneficial for muscle growth and immune function [23]. In truth, the protein content in insects is higher than in soya. For instance, crickets have an average protein content of about 65 g/100 g dry matter, outperforming other sources such as beef, eggs, milk, and soya [24,25]. Histidine, isoleucine, leucine, lysine, threonine, tryptophan, and valine are all essential amino acids found in cricket protein. These amino acids are necessary for the body and cricket protein contains enough of them to meet or exceed the recommended intake for adults [26]. Biological value of protein refers to the portion of protein that can be stored and utilized by the body after digestion and absorption. Edible insects, including grasshoppers (*Melanoplus foedus*), crickets (*Gryllus assimilis*), moths (*Cirina forda*), and termites (*Macrotermes nigeriensis*), typically have higher biological values compared to traditional protein sources. The biological value of these insects ranges from 85% to 93%. In comparison, the biological value of milk casein is around 73% [27].

The first challenge in utilizing insect proteins themselves is how to extract and isolate them. The protein content in insects is wealthy, usually between 50% and 70% after drying, but the extraction process will affect its content [28]. The alkaline extraction method is the most frequently used approach for extracting proteins from insects, where the proteins are precipitated at their isoelectric point by adjusting the pH of the insect slurry. This method is simple, cost-effective, and can be easily scaled up for industrial production. However, the yield of protein obtained by this method may vary depending on the insect species, and additional steps are required to achieve higher purity proteins. The protein content of mealworm larvae acquired through alkaline extraction and isoelectric precipitation at pH 4 augmented by 14.4% compared to the initial extract [29]. The characterization of proteins from locusts, crickets and silkworm pupae extracted by acid-alkaline precipitation has been studied, and the results show that this method effectively separates proteins from insects with little detrimental effect on their nutrition and function [30]. Another method for extracting insect proteins is ultrasound. Ultrasonic treatment resulted in 89%, 28%, and 34% increase in protein extraction from silkworm pupae, yellow mealworms and crickets, respectively, with no significant change in amino acid profile [31]. Ultrasound extraction boosts the yield of proteins and also causes structural changes through physical and chemical processes. This modification can alter the allergens’ epitopes, making them less accessible to the receptors of antibodies [32,33,34].

### 2.2. Insect Lipids

Insect lipids are the second most abundant group of insect nutrients, usually higher in the larval stage, with dried insects containing 10–50% lipids [35]. For example, beetles (*Rhynchophorus palmarum*) have a fat content of between 35–65% in the larval stage. However, as they enter adulthood, their fat content decreases [36]. The extraction of insect fats has little effect on the fatty acid composition of the extracts but has a larger impact on the extraction rate [15]. Insect larvae such as mealworms, waxworms, and fly maggots accumulate lipids as they grow. In addition, insect pupae, including silkworm and fly pupae, may also be a source of fat. Pupae usually have a richer fat content than other insect developmental stages. Study on the nutritional composition of honey bees by Ghosh et al. showed that fat content decreased with developmental stage [37]. Although a few adult insects, such as crickets and blackflies, can also be used for fat extraction, their fat content is usually lower than that of larvae and pupae [38].

Insect fats contain a high proportion of polyunsaturated fatty acids, particularly omega-3 and omega-6. These fatty acids have a positive impact on heart health and brain function [39,40]. The fatty acid composition and content differ among different insect species. For instance, in beetle larvae, oleic acid is the dominant fatty acid, while linoleic acid is dominant in cricket larvae. Additionally, the larvae of *Allomyrina dichotoma* and *Protaetia brevitarsis* have higher levels of palmitic acid. On the other hand, *Tenebrio molitor* larvae and crickets have higher linoleic acid content [11]. Insect fats are often referred to as insect oil because they are liquid at room temperature. They are rich in linoleic and linolenic acids, which give them a liquid consistency that makes them suitable as a food lubricant. They are high in unsaturated fatty acids, which make up about 57–75% of insect fat, and are useful for making pasta, sweets, and butter [41].

### 2.3. Insect Vitamins and Minerals

Vitamins and minerals are essential for maintaining proper body function and overall health [42]. While traditional food sources like vegetables, fruits, meats, and grains are commonly known as the main providers of these nutrients, edible insects are now recognized as valuable sources as well. Insects contain key vitamins such as B12, A, and D, as well as minerals like potassium, calcium, magnesium, zinc, iron, phosphorus, copper, and manganese. These nutrients can be extracted from insects and used as dietary supplements [43].

In general, insects typically have higher amounts of calcium, copper, zinc, and manganese than chicken, pork and beef [44]. *Oecophylla smaragdina* and *Odontotermes* are rich in iron, zinc, and copper, and proper consumption of these insects helps to meet the recommended daily intake [45]. Additionally, minerals in edible insects are easily absorbed and dissolved, similar to how beef tenderloin is utilized by the body [27]. Iron and zinc play the key roles in preventing malnutrition and early developmental delays [46,47]. However, insects are not receiving much attention as a source of iron and zinc in the human diet. Iron and zinc levels in edible insects can vary according to the species, stage of development, and substrate, ranging from 4–62 mg/100 g dry matter for iron and 9–27 mg/100 g dry matter for zinc [48]. Common vitamins found in insects are vitamin B12, riboflavin and thiamin, the levels of which can be affected by different species. Vitamin B12, which is primarily found in animal foods, is essential for individuals who do not consume meat or fish [49]. Consuming insects provides these essential vitamins that help maintain a healthy nervous system and red blood cells. For example, dried cricket powder has ten times more vitamin B12 compared to beef [43].

### 2.4. Other Compounds

Insect exoskeletons are a good source of chitin. Chitin is a natural, non-toxic polysaccharide with antimicrobial and antioxidant properties, and common sources include crustaceans, insects, and fungi [50]. The human gastric juice contains chitinase, which can digest chitin, providing evidence that humans can utilize chitin [51]. In the past, marine crustaceans have been the primary source of chitin. However, the cost of obtaining them is high [52]. An alternative source of chitin is the exoskeletons of insects, which are rich in chitin and are more easily reproduced and grown than crustaceans. Cricket chitosan has been shown to inhibit or eliminate pathogenic bacteria by promoting probiotic levels [53]. Unlike crustaceans, chitin from insects is usually extracted under less harsh conditions. Insects that are particularly rich in chitin include Coleoptera, Lepidoptera, Hymenoptera, Diptera, Orthoptera, and Hemiptera [54]. For example, the lepidopteran *Bombyx mori* produces silk fibers that mainly consist of sericin proteins, and the cocoons also contain chitin as a support material. Chitin can be obtained as a by-product of removing the sericin proteins from the cocoons during the silk production process [55]. In addition, insect residues such as wings, legs, or bodies discarded during processing can also be potential sources of chitin. These residues can be used to collect and processed to extract chitin, allowing for a more complete utilization of insects [56]. Notably, chitin extraction and purification methods may vary depending on the source and intended application. Commonly used techniques include deproteinization and demineralization processes, which remove proteins and minerals respectively, resulting in purified chitin [54].

Phenolic compounds have been recognized for their positive impact on human health. While plants are commonly known as a source of phenols, it is interesting to note that insects are also capable of producing these compounds [15,57]. Phenolic compounds, such as phenolic acids, flavonoids, and tannins, have antimicrobial and antioxidant properties and consist of a core structural component with a benzene ring and at least one hydroxyl group [58,59]. Insects have the ability to convert food into phenolics more efficiently than typical plants, requiring fewer resources such as water, land, and fertilizer. Furthermore, insect-sourced polyphenols are considered to be more sustainable than traditional plant-sourced polyphenols. Many insects, including bees, houseflies, yellow jackets, and crickets, provide phenolic chemicals [60]. Notably, the amount of polyphenols in insects varies depending on their diet and life stage [15,57,61,62].

Insect antimicrobial peptides (AMPs) are short proteins that have an immunological effect against bacterial, viral, fungal or parasitic organisms, and these antimicrobial peptides have potential applications in medicine and agriculture to address the challenges of antibiotic resistance [63,64]. Insects rely on these AMPs for their innate immune defense. Their diverse mechanisms of action, ability to target a wide range of microorganisms, and low likelihood of developing resistance make them ideal candidates for developing new antimicrobial drugs and medical interventions [65,66]. Antimicrobial peptides of insect origin have been classified into three categories: linear α-helical cecropins, defensins, and peptides rich in glycine and proline residues. *Bombyx mori* produces a series of antimicrobial peptides, including moricin, gloverin, and cecropin, which have broad-spectrum antimicrobial activity against bacteria, fungi, and even some viruses [67]. Cecropin, the first insect antimicrobial peptide discovered, was found in pupae of *Hyalophora cecropia* [68,69]. Cecropin inhibits the growth of viruses and parasites associated with malaria [70]. Maggots from the green bottle fly (*Lucilia sericata*) produce antimicrobial peptides, including fluorescein and filipin, which have potent antimicrobial properties and can help in wound healing [71,72].

The beneficial functions of certain active compounds in edible insects have been recognized by the general public. However, anti-nutrients may be of some controversy. Edible insects mainly feed on plants. Plants synthesize different types of secondary metabolites for self-protection during the growth process, and these substances accumulate in the insect’s body as the insect feeds on the plant. Consequently, this accumulation hampers the insect’s ability to absorb vital nutrients. Therefore, these substances are called anti-nutrients. The anti-nutrients in the insect’s body vary depending on the plant consumed and mainly include phytate, tannin, oxalate, trypsin inhibitors, lectin, and hydrocyanide [73]. Anti-nutrients have beneficial effects in the treatment of certain diseases, especially in the treatment of obesity, diabetes, antiviral therapy, neuroprotection, lowering of blood cholesterol levels, and inhibition of cancer [74]. Therefore, anti-nutrients obtained from edible insects may be a potential method of preventing the above related.

## 3. Production and Utilization of Edible Insects

The development of insect farming and processing technologies is necessary to facilitate the promotion of insects. Here, we discuss current trends and advanced methods of insect farming, as well as the impact of different processing techniques on the final product. We explore various applications and potential future trends for edible insect products, providing valuable insights into the potential and possibilities of edible insects as a sustainable food resource.

### 3.1. Development of Insect Farming Technology

Using insects as a sustainable food source has led to significant advancements in insect rearing techniques. These technologies aim to increase yields, lower costs, enhance quality, and promote the sustainability of the production process [75]. Insects are susceptible to environmental conditions, including temperature, humidity, and light. Rather than relying on wild collection, which is both inefficient and disrupts wildlife ecosystems, intensive farming systems have been developed to farm insects on a large scale in controlled environments [76]. Modern insect breeding system creates the best growth conditions for insects by adjusting factors such as temperature, humidity, and light to ensure that environmental factors no longer hinder the growth of insects and improve the growth and maturity speed of insects [77,78]. What’s more, the nutritional composition of insects is influenced by their feed, so scientists have developed insect feed adapted to the growth of insects to ensure that they get enough nutrition [79,80]. This artificial feeding method ensures that insects get enough food at different growth stages, thus improving productivity. The insect breeding mode improves production efficiency, reduces the dependence on wild insect resources, and ensures the sustainable supply of insects as food sources.

According to a review of studies on the East African edible insect farming industry, insect farming is a very new industry in East Africa, but many farmers and businesses are entering the field. In East Africa, there are several enterprises involved in black soldier fly farming, with the ninth-ranked company producing enough insect protein to feed approximately 4.7 million chickens per year. Additionally, this company also generates waste that can be utilized as agricultural fertilizer [81].

*Vespa mandarinia* is rich in unsaturated fatty acids and minerals, making it a nutritious and delicious food [82]. There are well-established *V. mandarinia* farming industries in China, Korea, and India. Previously, *V. mandarinia* was usually caught in the forest, a direct harvesting method that could destroy the habitat and threaten its survival. As its taste and nutritional qualities have become more recognized, *V. mandarinia* has been gradually semi-domesticated and is being farmed in captivity, which not only protects its biodiversity but also increases its production [83].

It is worth noting that when farming insects in temperate countries, we need to be aware that maintaining a suitable environment for insect growth requires attention to electricity and water consumption, cleaning and disinfection, microbial control, and insect escape, all of which require capital for production. So even though the safety risk of eating insects is significantly reduced with large-scale farming, at the same time, the investment in captive breeding costs will make insect production more expensive, and insects may no longer be a very cheap source of food, which may not be conducive to the promotion of insects. The future direction of insect farming should be more sustainable and economical, utilizing organic waste as much as legally possible, reducing production costs while at the same time protecting the environment and reducing the production of greenhouse gases.

### 3.2. Processing Technology of Edible Insects

The processing of edible insects is crucial to the quality and safety of the end product. This involves a series of stages, starting from the capturing or farming of the insects, followed by harvesting, killing, handling, and processing [84]. The advancement in processing technologies plays a vital role in maintaining food safety standards through efficient production lines and strict adherence to cleanliness protocols. Once the insects are collected or farmed, appropriate methods must be employed to kill them in order to prevent the spread of bacteria and parasites. These processing techniques include freezing, boiling water treatment, hot air drying, and steam treatment [85]. After killing, insects need to be cleaned and skinned to remove unwanted parts and impurities [86]. Subsequently, the insects can be cooked and processed according to the specific requirements of the food. Cooking methods such as frying, roasting, smoking, deep-frying, and preparing soups can be employed. Additionally, insects can be processed into powdered form, granules, or paste to create food products like protein bars, energy bars, and pasta [87]. In addition to food processing, insects can be extracted to make food additives and functional ingredients. These ingredients can improve the nutrition and functionality of other foods. However, it is worth noting that different processing methods can impact the nutritional value and the characteristics of the final product of the edible insect. Research has shown that improper heat treatments like boiling, baking, and frying can damage nutrients and active chemicals in insects [88]. In a study conducted on *Tessaratoma papillosa*, a common edible insect in Thailand, it was found that baking and heat treatment increased the phenolic acid, tocopherol, cinnamic acid, and amino acid contents, while decreasing the fiber content [89]. To prevent this, new processing techniques have been invented in recent years, such as fermentation, hydrolysis, extrusion, supercritical extraction, and curing [85]. Each of these technologies offers advantages over traditional processing methods, and it is important to select the appropriate technique according to specific processing requirements. Table 2 provides an overview of commonly used processing techniques for insects and their applications.

In the processing of insect foods, food safety is a critical concern. Ensuring hygiene in processing, avoiding cross-contamination, and proper cooking are essential measures to ensure insect food is safe. In addition, it is necessary to adhere to food labeling and regulatory requirements to ensure that insect foods are legally sold.

In summary, the ongoing advancements in insect farming and food processing technologies are laying a strong foundation for the sustainability of the insect food industry. These technological improvements not only enhance the efficiency and quality of insect foods but also reduce reliance on traditional food sources, which opens new possibilities for the future food supply chain.

### 3.3. Edible Insect Products

Eating insects has a long history in many cultures. In recent times, there has been an increasing acceptance and exploration of insect foods, such as cricket meal, silkworm pupae, and grasshoppers. There are an estimated 2111 species of edible insects worldwide, belonging to different species and genera [99,100,101]. These insects are mainly found in the Coleoptera, Orthoptera, Lepidoptera, Diptera, Hymenoptera, Hemiptera, Isoptera, and Diptera orders, with Coleoptera being the most abundant [18,102,103]. Insect foods offer a wide range of nutritional benefits and also possess unique tastes and flavors. Additionally, some insects are used as feed for animals, indirectly contributing to the human food supply.

#### 3.3.1. Traditional Snacks

Edible insects have been a source of food in various traditional cultures and regions, with bees, wasps, locusts, grasshoppers, crickets, silkworms, bamboo caterpillars, beetles, ants, termites, and flies being the most consumed insect groups. Across different cultures and regions, there is a rich diversity of traditional insect snacks and other arthropod. These snacks are often influenced by local ingredient availability and cultural context. For example, fried scorpions and grasshoppers are common street foods in Southeast Asia, while fried grasshoppers are popular foods in Africa, as well as fried scorpions in Thailand and grasshoppers in Mexico [104]. Traditional insect snacks are prepared in a variety of ways. The insects are typically cleaned, cooked, seasoned, and then processed into snacks by frying, grilling, fumigating, or deep-frying. These preparation methods make the insects crispy and appealing to the consumer’s appetite [96]. Insects can be consumed at various developmental stages, including eggs, larvae, pupae, and adults. However, larvae and pupae are the forms that are most frequently consumed. Insectivory has been a part of many cultures throughout history, and we have compiled a list of popular edible insects and how they were consumed worldwide (Table 3).

Although traditional insect snacks face challenges in the modern food market, such as changing consumer attitudes and food regulations, they still hold historical and cultural significance. Additionally, their ability to support sustainable food production makes them relevant and suggests they may have a role in the future of the food sector.

#### 3.3.2. Insect-Derived Foods

Although researchers recognize edible insects as a source of good nutrients, the development of edible insects is limited by consumer acceptance due to many people’s natural fear of insects. More consumers prefer to consume insect-derived foods rather than eating them directly [117]. In addition to directly eating insects through traditional processing methods, innovative food products that utilize insect raw materials, such as energy bars made from insect protein and bread processed from insect powder, are becoming more popular [87,96]. The most popular eating insects are mealworms, grasshoppers, and crickets. They are processed into delicious snacks used as nutritional supplements for sports in addition to being consumed directly [84].

People are more inclined to accept edible insect products than eating the insects themselves. To boost the nutritional content of foods without altering their texture or flavor, insects are processed into a variety of forms, including pastes, powders, and meals. These components are then used in cooking and baking items [118]. For example, insect powder can be mixed with flour to make pasta, bread, burgers, and biscuits. This practice not only helps reduce the aversion to insect food but also increases the protein and fat content of these products [119,120,121,122]. Insect proteins might be utilized in the industry of food and beverage as protein supplements, such as cricket meals or mealworm meals, which can be used as an ecologically friendly substitute for protein in protein bars, shakes, and other dietary supplements [123,124]. Not only can insects be made into functional foods, they can also be used to develop plant-based or hybridized meat substitutes that provide sustainable, protein-rich ingredients. A study in which samples of *Pterophylla beltrani* were added in varying proportions during tortilla preparation increased the content of phenolic and antioxidant compounds in tortillas, suggesting that high-temperature treatments (100–115 °C) do not destabilize the active substances [125]. Adding insects such as *Schistocerca piceifrons*, *T. molitor* and *P. beltrani* to produce alcoholic beverages enables them to contain more phenolic and antioxidant compounds, which are also relatively stable over time at room temperature [126,127,128]. Research has shown that insect oil, obtained from silkworm pupae using organic acids such as acetate and citrate, contains a wealth of minerals and beneficial fatty acids, including linoleic and linolenic [129]. The levels of heavy metals and toxic elements in the extracted insect oil are negligible, making it suitable for human consumption.

#### 3.3.3. Insect Feed

Promoting insect use as animal feed is an alternative method of utilizing them. Insect feed has a much higher conversion efficiency compared to traditional feed for chickens, cows, and pigs [130]. People already consume traditional livestock that are fed insects, such as pigs, chickens, and fish. Indirectly consuming insects through animal consumption is a more accepted way to incorporate them into our diet. Insects constitute a natural food source for a range of animals, including but not limited to chickens, ducks, poultry, and fish. Animals fed with insect-based feed have similar or even better quality compared to those fed with traditional feed. The exclusive antimicrobial peptides and other substances found in insect feed have the potential to bolster animals’ immunity to diseases [131].

Mealworms, grasshoppers, cockroaches, and black soldier fly larvae are the most used in insect diets. Black soldier fly (*Hermetia illucens*) larvae are renowned for producing high-quality protein, making them a sought-after substance to use in animal feed. There were no negative effects on the growth of rabbits when soybean meal was replaced by two insect feeds (crickets and yellow mealworm meal) [132].

Fishmeal and fish oil have traditionally been used as the main sources of protein in fish feeds for aquaculture. However, due to the declining availability of wild marine fish resources, their use has become unsustainable. As a result, vegetable proteins and oils have emerged as viable alternatives [133,134,135]. Unfortunately, plant-based feeds contain certain substances known as anti-nutrients, which can negatively impact fish consumption, digestion, and nutrient absorption. This can lead to slower fish growth and reduced resistance to diseases [136]. The beneficial effects of the high chitin content found in the nymphal exuviae of *H. illucens* on fish intestinal flora have been demonstrated [137]. The impact of substituting fishmeal with *H. illucens* larval insect meal on the growth of Whiteleg Shrimp was investigated by Hermes et al. [138]. Notably, Whiteleg Shrimp exhibited a greater survival rate when fed with the insect meal. This improvement could potentially be attributed to the presence of certain antimicrobial compounds found in the insect meal, which contribute to the enhanced survival of Whiteleg Shrimp.

In addition to being used as livestock feed, insects can be added to pet feeds, such as cats, dogs, snakes, frogs, and beetles. The utilization of insects as animal feed and feed additives decreases their reliance on other protein sources, leading to an indirect advantage for human beings. Traditional commercial pet food production generates significant amounts of methane and nitrous oxide, which have a negative impact on the environment [139]. As mentioned, rearing insects produces significantly fewer greenhouse gases compared to traditional poultry production, making the adoption of insects as an alternative to standard pet food beneficial for the environment. Giant mealworms, which are larger neotropical beetles, serve as a protein source for small domestic animals and are multiple times larger than regular mealworms. They have been utilized as a protein source for small pets like reptiles, birds, and small mammals [140]. Orthopteran insects, including locusts, crickets, and grasshoppers, are highly nutritional and frequently utilized as part of pet and zoo animal diets [141]. Table 4 provides a summary of research conducted on the inclusion of insects in pet food for dogs and cats.

#### 3.3.4. Food Additive

The utilization of insects as food additives is an emerging trend in the food industry. Insects offer distinct flavors, nutrition, and functionality that make them valuable in enhancing the taste of food. Different species of insects possess unique flavor profiles, such as nutty, seafood, or earthy flavors, which can be utilized to enhance the overall taste of dishes. Insect powder can also be utilized as a natural food colorant, imparting a distinctive color to food products [149]. Moreover, insect powder can improve the texture of foods and increase their taste. Insects are also rich in functional ingredients such as antioxidants, antibacterial agents, and dietary fiber. Extracting and incorporating these ingredients into food can enhance its freshness and nutritional value. *Tteokgalbi*, a meat product, was prepared by adding 2% A. dichotoma extract, which effectively prevented lipid oxidation and improved the quality of the meat product without impacting microbial abundance [150].

### 3.4. Potential Risks

There are also some risks associated with utilizing edible insects as a potential food source. We have summarized the possible risks of edible insects in the following four points. (1) the allergenicity of insects. Studies have reported a wide range of insect allergens, with proto-myosin being the most common [151]. Panallergic structures are present in bees, beetles, locusts, and cockroaches. (2) harmful microorganisms [152]. Natural insects themselves grow in a variety of environments, and some of them feed on animal carcasses and organic wastes and even discriminate, so they may carry a large number of microorganisms on the surface of their bodies and in their bodies, which can only be safely consumed when they are correctly processed and stored [153,154]. (3) toxic metal elements. These substances accumulate throughout the insect’s life due to different factors such as species, developmental stage, and feed substrate. Arsenic and cadmium have been found in yellow mealworm larvae, Bombay locusts, saint beetles, black gadflies, house crickets, and mulberry silkworms. (4) anti-nutrients, there may be some antinutrients present in the insects, as these can alter the bioavailability of proteins and minerals [73]. Oxalates, alkaloids, and saponins have been reported in some insect species (giant white ants and termites) [27]. These potential risks can prevent the utilization and development of insects, so a systematic solution is needed to control the generation of these risks, and insect-scale farming in controlled environments is a good solution.

## 4. Sustainability of Edible Insects

Insects have advantages over traditional animal husbandry when it comes to mitigating the effects of climate change and reducing our carbon footprint [155]. Crickets are four times more efficient than pigs in converting feed into “meat” [156]. Insects can be raised at high densities in a small space, which reduces the amount of land needed. Additionally, insects require less water compared to raising livestock and growing crops, which is important for addressing water scarcity issues. Insects provide another advantage through their capacity to consume organic waste, such as kitchen and agricultural waste, and convert it into valuable proteins and nutrients. This helps reduce the burden of waste disposal and decreases the need for landfilling and incineration [157].

Insects produce various types of waste, such as exuviae and feces, during their growth. These insect wastes can be utilized as fertilizer in the soil to improve the soil fertility and enhance its resistance to diseases [158]. Using insect waste as bio-organic fertilizer in agriculture circumvents the adverse environmental effects ensuing from chemical or synthetic fertilizers, such as soil acidification, nitrous oxide emissions, and water eutrophication [159,160]. The insect-based fertilizer recycles endogenous nitrogen (N) and phosphorus (P) from the food system and returns it to the food system, thereby preventing the build-up of excessive N and P in natural systems [161]. Insect excrement presents a fresh form of biologically organic fertilizer. The nutrients within insect waste possess the potential to stimulate plant development and heighten crop durability against illnesses [162]. The N, K, and P content of mealworm biofertilizer is similar to that of highly fertile poultry manure [163] Incorporating black soldier fly larvae excrement substantially elevated the pH levels, organic matter content, ammonium peptide nitrogen levels, phosphorus levels, and potassium levels in the soil of the rice plantation [164]. Furthermore, the presence of insect excrement reduced the uptake of weighty metals, specifically lead (Pb) and cadmium (Cd), by the rice plants. A comparative analysis performed by Beesigamukama et al. compared the effects of applying black soldier fly frass fertilizer (BSFFF) and commercial organic fertilizer (SAFI) on various aspects of soil health [165]. The results indicated that the BSFFF treatment resulted in a higher concentration of ammonium nitrogen in the soil, while the SAFI-treated soils had higher levels of nitrate nitrogen. Additionally, the application of BSFFF resulted in an increase in bacterial and fungal populations, a decrease in soil acidity, and an increase in phosphorus and magnesium release compared to the SAFI-treated soils.

Overall, insects as a food source have considerable environmental benefits in terms of sustainability. Insect foods are hopeful to play a more significant role in the future food supply chain. However, additional research and investment are necessary to foster more sustainable production and consumption practices and fully exploit the potential of insect sustainability

## 5. Market Acceptance and Challenges of Insect Foods

Insect foods have been consumed in certain areas for a long time, but their acceptance in the global market varies significantly. Gaining consumer recognition and acceptance remains the biggest challenge for insect consumption [166]. The attitudes of consumers towards insect foods are influenced by factors such as culture, customs, and personal beliefs. It is crucial to understand these differences in order to effectively market and promote insect foods. However, there are also legal and regulatory obstacles that insect foods face. The regulations for insect foods differ from country to country, which can complicate and create uncertainty in accessing the market for these products. In order to advance the insect food sector, it is crucial to address these legal and regulatory challenges. By doing so, we can gain a better understanding of the future trends and opportunities for insect food as a sustainable source of nutrition.

### 5.1. Consumer Acceptance

Consumer attitudes towards edible insects are influenced by factors such as region, food aversions, phobias, environmental awareness, health awareness, risk of insect consumption, personal experience, and culture [167,168]. Understanding why these factors arise can contribute to the improved promotion of insect foods. We classify these effects into three primary categories and analyze them within the framework of current research findings.

#### 5.1.1. Social Factors

Age, gender, and education have been extensively explored as influential social factors affecting the acceptance of insects among consumers. The majority of studies suggest that younger individuals tend to be more accepting of insects compared to older individuals, and those aged between 15 and 35 exhibit higher levels of acceptance compared to other age groups [167]. However, in regions with a history of consuming insects, the outcomes may be different. For instance, in China and Japan, older individuals who have experience with eating insects are more likely to embrace insect foods compared to younger individuals [168,169]. Therefore, it is essential to gather more definitive evidence on how age influences the acceptance of insects by consumers. In studies that examined the influence of gender on insect consumption, the majority of studies found that males displayed a higher degree of acceptance towards insects, while a few studies found no significant correlation between gender and acceptance of insect consumption [170]. Highly educated individuals might hold favorable attitudes towards the promotion of insect foods. However, certain studies suggest that there is no correlation between the level of education and the acceptance of insect foods [171,172]. Moreover, comparing educational achievements is challenging due to disparities in educational resources and levels across different regions and countries.

#### 5.1.2. Personal Emotional Factors

The level of individuals’ knowledge and comfort with regards to consuming insects as food, as well as their likelihood to develop new fears or aversions towards novel foods, are all key factors that affect their willingness to accept edible insects. A survey conducted to assess people’s awareness about the concept of edible insects and their understanding of the advantages associated with consuming such food revealed that increased familiarity had a notably stronger positive influence on the acceptance of edible insects [173]. The fear of trying new foods may also impede the acceptance of insect foods. However, research has demonstrated that most of the fear associated with new foods arises when a whole insect is used as an ingredient. This fear can be reduced when the insect is incorporated into the food in an invisible form [174]. Becoming more familiar with edible insects can reduce the negative impact of phobias towards new foods on the promotion of insect consumption [175]. Aversion refers to a negative consumer attitude towards edible insects. In ancient Rome, insects were consumed both as a delicacy and as a means of sustenance for individuals facing poverty during times of food shortage [174]. However, in Europe, insects are generally regarded as unclean and repulsive pests that people prefer to avoid or have nothing to do with [175]. Many individuals express resistance or concern about consuming insects because they may be concerned about potential allergies, microbial and parasitic risks, or have reservations due to insects’ appearance, taste, or psychological barriers [166,176,177] This aversion is deeply ingrained and not easily changed. Research indicates that people in Western countries primarily consume insects not for their taste, but for the unique advantages they offer, such as their high nutritional value [173]. Therefore, increasing the invisibility of insects in foods through processing and increasing awareness and education about the benefits of consuming insects can help to address this issue [176].

A recent study has shown that the preference for eating crickets among consumers can be increased by implementing interventions that emphasize descriptive social norms [177]. These interventions involve increasing the number of people and cultures that openly express their love for eating insects. This is because individuals tend to follow the crowd when they are unsure about making choices. However, it is worth considering that this approach may not be effective for populations that have a strong aversion to insects.

#### 5.1.3. Dietary Factors

Consumers’ dietary preferences and their knowledge of green diets can influence their choice of insect foods [178]. Vegetarians are very reluctant to include insects in their diets [149,179]. Meat lovers were more likely to accept insects, but meat lovers who preferred traditional meat were less likely to accept insects as a substitute for meat [180,181]. People with traditional food cultures also have a low willingness to accept insect foods. For instance, individuals in southern Italy, where the Mediterranean food culture is predominant, generally exhibit hesitance towards incorporating insects into their dietary practices [182]. Individuals who have previously consumed insects and have developed a favorable perception of them are more inclined to embrace insect foods. Hence, the initial sensory experience of an insect food plays a crucial role in determining its acceptance. Elevating the flavor profile of insect products can effectively create a positive impression among consumers, thus contributing to the increased acceptance of these food products [183]. Most studies have shown that people who have a green diet and are aware of the environmental benefits of consuming insects have a higher acceptance of insect foods, and utilizing consumers’ environmental awareness is also an important means of increasing insect consumption [182]. Therefore, there is a need for education and awareness-raising campaigns to help people understand the benefits and sustainability of consuming insect foods in order to increase consumer acceptance of insect foods.

### 5.2. Legal and Regulatory Issues

Establishing a sound regulatory framework is crucial to guarantee the quality and safety of insect food products. The lack of clear legislation is a severe constraint on developing edible insects. The global attitude towards edible insects is highly valued. However, there is significant variation in legislation from one country to another. Among these countries, the European Union (EU) has the most comprehensive and continuously improving regulations for edible insects. In 2018, the new EU Food Regulation (EU) 2015/2283 came into force, which classified edible insects as farmed animals, *Locusta migratoria* adult, *Alphitobius diaperinus* larva, *Acheta domesticus* adult, *and Tenebrio molitor* larva were approved for use in insect food. Insect food products must meet the same regulatory requirements as conventional food products before they can be sold in the market. This includes undergoing food approval procedures and complying with labeling requirements. EU Member States are responsible for ensuring that insect food products adhere to these regulations [184,185]. Regarding the utilization of insects as feed for farmed animals, EU regulations permit the addition of insects to aquaculture, poultry, and pig farming [186]. The application of insects in animal feed is subject to regulations through (EU) 2017/893 and (EU) 2021/1925. These regulations outline that exclusively eight insect species are presently permitted for use in animal feed within the EU. These species include the blackfly (*Hermetia illucens*), the common housefly (*Musca domestica*), the yellow mealworm (*Tenbrio molitor*), the small mealworm (*Alphitobius diaperinus*), house cricket (*Acheta domesticus*), banded cricket (*Gryllodes sigillatus*), crickets (*Grillus assimilis*), and silkworms (*Bombyx mori*) [187]. The EU is currently developing regulations for maintaining sustainability in insect farming, with a focus on factors like land use, water management, and ecological balance to ensure that the farming process does not have a negative impact on ecosystems [184].

In the United States and Canada, food safety regulations are overseen by the Food and Drug Administration (FDA) and the Food Inspection Agency (CFIA), respectively. The U.S. Food, Drug, and Cosmetic Act of 2013 indicates that insects are classified as food and subject to food regulations, including health and safety standards. When insects are utilized as an ingredient for food, rather than being marketed as food itself, obtaining a food additive license becomes mandatory. The FDA approves *H. illucens* larvae as an ingredient for animal food in livestock feed, while *H. illucens* larvae can be raised on approved organic waste streams. Canada states that a food is not new if it has a history of safe consumption internationally. Although there are no specific regulations for insect foods in Canada, insect foods are still subject to the general food regulations established by the CFIA [188]. Additionally, pet snacks consisting of black soldier fly larvae, mealworms, and silkworm pupae are sold in Canada.

Africa, a region with a rich tradition of insectivory, has very little legislation relating to insect food, with Botswana being the only country in Africa that currently has provisions for the consumption of insects in its legislation, while most other countries do not have explicit provisions for this. In the absence of explicit legislation, insects are allowed to be considered as food in most parts of Africa and are claimed under general food laws [189]. Some countries in Africa categorize some edible insects as protected animals, so in these regions, such insects are not allowed to be consumed, such as the Cape Verdean beetle. Mealworms and locusts can be eaten, but when they appear in large numbers as agricultural pests, they cause huge economic and food losses. The relevant African agricultural management committees have taken several actions to curb the proliferation of agricultural pests but have no intention of treating them as a food resource, largely because of a lack of regulations.

Asia, China, Japan, and Thailand have a rich history of consuming insects. As of 2023, the Catalogue of Edible Insect Resources issued by China’s National Health and Health Commission has approved 26 species of insects as new resources. However, insects are currently not included in China’s food catalog lists for its citizens due to various challenges such as market access, dietary concepts, public opinion campaigns, lack of education, and limited large-scale industrial support. Although the tradition of eating insects exists in Japan, the number of people who eat insects is small, and regulations regarding it still need to be developed [26]. Edible insects are everywhere in Thailand, where Good Agricultural Practice standards were created and widely used for insect farming [190].

In summary, the insect food market faces challenges such as consumer attitudes, cultural differences, and complex legal and regulatory issues. To tap into the potential of the insect food market, we need to change consumer attitudes through education and awareness campaigns and establish an appropriate regulatory framework to guarantee the safety, quality, and sustainability of insect-based consumables. To promote a healthy insect food market, it is crucial for governments, industry, and consumers to collaborate closely.

## 6. Conclusions

As individuals become more conscious of the environmental impacts of food production, incorporating insects into our diets will be seen as a means to reduce greenhouse gas emissions and minimize resource waste. The edible insect industry is currently in its early stages of progress, but there is substantial potential for technological advancements and market expansion. Governments and international organizations may implement additional measures to encourage the production and consumption of food based on insects. Additionally, regulators must establish clear guidelines to address various aspects related to insect food, including safety, regulations, standards, and production efficiency. In summary, edible insects possess significant promise as a developing food source. Nevertheless, accomplishing this objective necessitates collaboration and persistent endeavors from all stakeholders.

## Figures and Tables

**Table 1:** Comparison of nutrient content between common insects and conventional poultry [17].

Source	Protein (g/100 g)	Fat (g/100 g)	Saturated Fat (g/100 g)	Sodium (mg/100 g)	Calcium (mg/100 g)	Iron (mg/100 g)	Lodine (mg/100 g)	Vitamin C (mg/100 g)	Vitamin A (mg/100 g)	Riboflavin (mg/100 g)	Niacin (mg/100 g)
Beef	20.6	9.3	3.8	60	5	1.95	10	0	0	0.23	4.7
chicken	19.9	7.2	1.81	80	8	0.88	6	1.1	0	0.16	6.5
pork	20.1	12.4	3.5	62	7	0.8	5	0	0	0.235	5.6
Cricket (adult)	20.1	5.06	2.28	152	104	5.46	0.021	3	6.53	3.41	3.84
Honeybee (brood)	15.2	3.64	2.75	19.4	30	18.5	NA	10.5	25.7	3.24	NA
Silkworm (pupae)	14.8	8.26	3.45	14	42	1.8	NA	NA	NA	1.05	0.9
*Mopane caterpillar* (final instar)	35.2	15.2	5.74	NA	700	NA	NA	NA	NA	NA	NA
*Palm weevil* (larvae)	9.96	25.3	9.84	11	39.6	2.58	NA	0.00425	11.3	2.21	NA
Mealworm (larvae)	19.4	12.3	2.93	53.7	42.9	1.87	0.017	1.2	9.59	0.81	4.07

NA = not available.

**Table 2:** Insect processing techniques and their scope of application.

Processing Technology	Goal	Scope of Application	References
Boiling, grilling, frying, deep-frying, and smoking, curing	Cooking insects for consumption and extending their shelf life	Traditional cuisine	[90]
Scalding (steam or boiling), pasteurization	Reduces degradation and destruction of products by enzymes, reduces microbial contamination, inactivates food, inactivates endogenous insect enzymes	Inactivation of enzymes and destruction of pathogenic bacteria	[84,91]
Drying (freeze-drying, heat-drying)	Reduces moisture, reduces microorganisms, extends shelf life	Preparation of edible insect powder	[92,93]
Soxhlet Continuous Extraction, Three-Phase Fractionation, and Supercritical Carbon Dioxide Extraction	Separation of insect lipids and fats	Extraction of insect lipids	[94,95]
Hydrolysis	Enzymes, acids, or bases hydrolyzed insects for subsequent extraction of insect proteins.	Insect protein extraction	[96,97]
Fermentation	Improved nutritional quality and flavor	Fermented insect soy sauce, biofuel, feedstuffs	[85,98]

**Table 3:** Different orders of common edible insects and their traditional ways of cooking.

Insect Order	Typical Insect	Cooking Method	References
Coleoptera	*Palm weevil* (*Rhynchophorus ferrugineus* larva)	Eat raw, boil, fry, grill	[105,106]
	bamboo worm (*Omphisa fuscidentalis*)	deep-fried	
	huhu beetle (*Prionoplus reticularis*)	Eaten raw or fried, it tastes like peanut butter	[107]
	mealworm (*Tenebrio molitor*)	Made into insect snacks and insect oil	[108]
Hymenoptera	ant (*Pheidole megacephala*)	Roast or serve as a sweet dish	[109,110]
	honey bee (*Bee*) and wasps (*Wasp*)	Collect honey, larvae and pupae for roasting and frying	[109]
Orthoptera	cricket (*Acheta domesticus*)	Eat whole by cooking or make into snacks	[111]
	Grasshoppers (*Sphenarium purpurascens*) and grasshopper (*locusts*)	Roast, fry, make soup or stew	[112,113]
Isoptera	Termite (*Macrotermes bellicosus*)	Grilled or fried for a crispy texture	[105]
Hemiptera	cicada (*Cicadidae*)	boil or fry	[114]
	stinkbug (*Encosternumdelgorguei*)	grate into paste	[115]
Lepidoptera	caterpillars (*Clanis bilineata*) and silkworms (*Bombyx mori*)	Fried, baked, boiled; worm tea (made from eating the feces excreted by certain types of plants, mostly consumed in China)	[116]

**Table 4:** Insects in pet cat and dog feeds.

Insect Species	Pet	Finds	References
*Tenebrio molitor* larvae	Cat	Different species of pets have different preferences for each insect, with cats preferring *T. molitor* and dogs preferring *H. illucens*	[142]
	dog	Insect feed can entice dogs to eat, but gender affects it	[143]
*Hermetia illucen* larvae	Cat	*H. illucens*, which is suitable for cats, is basically suitable for other animals.	[144]
	beagle dog	Substituting *H. illucens* larval fat for chicken fat in the original feed does not affect the palatability of the feed to dogs.	[145]
	dog	The addition of relatively small amounts of *Hermetia illucens* larval feed significantly improved digestibility and had a beneficial effect on immune and antioxidant status.	[146]
*Musca domestica* larvae	Beagle dog	Administration of *Musca domestica* at the 5% level does not affect the dog’s growth, feed intake, hematology, biochemistry or immune properties, nor does it reduce oxidative stress.	[147]
*Shelfordella lateralis* (adult)	Dog	Insect feed can lure dogs to eat, but gender will make their favorite insects different.	[143]
*Gryllodes siggilatus* (adult)	Beagle dog	After adding 24% *Gryllodes siggilatus* to the beagle’s diet, the total microbial community in the beagle’s body was unaffected.	[148]

## Data Availability

Data are contained within the article.
